# Role of Dual-Contingency Management in Family-Based Obesity Therapy and the Effects of Weight Loss on Liver Transient Elastography Parameters in Youth: A Pilot Study

**DOI:** 10.7759/cureus.36629

**Published:** 2023-03-24

**Authors:** Indrajit Majumdar, Andrew H Talal, Carrol M Harmon, Emily Tabaczynsk, Kristen Cercone, Brian H Wrotniak, Lucy D Mastrandrea, Teresa Quattrin

**Affiliations:** 1 Pediatric Endocrinology, Mount Sinai Medical Center, New York, USA; 2 Pediatric Endocrinology, Valley Medical Group, Paramus, USA; 3 Medicine, Division of Gastroenterology, Hepatology and Nutrition, Jacobs School of Medicine and Biomedical Sciences, University at Buffalo, The State University of New York, Buffalo, USA; 4 Surgery, Pediatric Surgery, John R Oishei Children's Hospital/Jacobs School of Medicine and Biomedical Sciences, University at Buffalo, The State University of New York, Buffalo, USA; 5 Pediatrics, Roswell Park Comprehensive Cancer Center, Buffalo, USA; 6 Psychiatry, John R. Oishei Children’s Hospital/Jacobs School of Medicine and Biomedical Sciences, University at Buffalo, The State University of New York, Buffalo, USA; 7 Pediatrics, John R. Oishei Children’s Hospital/UBMD Pediatrics, Buffalo, USA; 8 Pediatrics, Division of Pediatric Endocrinology and Diabetes, John R. Oishei Children's Hospital/Jacobs School of Medicine and Biomedical Sciences, University at Buffalo, The State University of New York, Buffalo, USA; 9 Pediatrics, Division of Pediatric Endocrinology and Diabetes, Jacobs School of Medicine and Biomedical Sciences, University at Buffalo, The State University of New York, Buffalo, USA

**Keywords:** pilot study, liver transient elastography, fatty liver disease, contingency management, youth, obesity

## Abstract

The pilot study evaluated contingency management (CM) for family-based obesity therapy (FBT). The secondary outcome assessed the association of the hepatic transient electrography (TE) parameters, including the controlled attenuation parameter (CAP) and liver stiffness (LSM), and changes in liver function blood tests and BMI changes in youth involved in intensive FBT. It included youth-parent dyads from an urban pediatric center randomized to weekly behavioral therapy (BT, n= 4) who received fixed financial compensation for attendance, or BT+CM (n= 5) who received an escalating monetary reward for weight loss. At week 30, all youth and parents had weight-loss trends without significant differences between groups. While the TE measures and blood tests were normal in the youth at baseline and week 30, the CAP changes correlated with BMI changes (R^2^= 0.86, P< 0.001) and LSM changes with alanine aminotransferase changes (R^2^= 0.79, P=0.005). In conclusion, BT+CM did not significantly add to the BMI improvement seen with BT alone in youth and their parents. However, in youth with obesity and normal liver blood tests, TE may be useful for monitoring changes in fatty liver disease.

## Introduction

Obesity in adolescence is a significant public health problem, affecting approximately 19% of the population [1]. Behavioral modification remains the cornerstone for successful weight loss efforts, especially in youth where weight loss medications and surgical therapies have had limited application. Many interventions targeting obesity focus on the family-based simultaneous treatment of the parent and child [2]. Adherence to a weight loss program is essential for success [3]. Commitment generally wanes as weight loss becomes more challenging and families are referred to higher levels of care requiring frequent patient-provider contact. However, practical difficulties, such as transportation access, poor motivation, and social support, often interrupt treatment [4]. Without significant ongoing incentives, weight management strategies, including multi-disciplinary programs, face high attrition [3, 5], despite improvements in weight outcomes [6]. These challenges have been compared appropriately to substance abuse treatment [7].

One approach that has been successful for clinical conditions with high recidivism, such as substance abuse, is contingency management (CM) [8], in which individuals with substance abuse disorders are reinforced for abstinence with monetary rewards [9]. CM programs typically pair frequent urine drug screens with contingent, escalating awards. This strategy has had a remarkable success that lasts beyond the duration of treatment [8]. Due to the recalcitrant nature of weight loss in obesity, investigators have used CM for obesity treatment with initial success [10]. In addition, CM has demonstrated promise when added to short-term, home-based adolescent weight loss programs [11]. However, no studies have assessed the effectiveness of a simultaneous parent-youth CM treatment approach.

To address this gap, we proposed a novel dual CM strategy that combines simultaneous parent-child treatment with operant conditioning techniques of CM. Parent-child family-based behavioral therapy (BT) is a well-established strategy superior to individual therapy [2]. We included youth and at least one parent with obesity who prospectively participated in an urban tertiary weight management program for 30 weeks.

The primary study aim was to compare changes in body mass index z-score (zBMI, adolescent) and BMI (parent) for families randomized to the BT+CM versus the non-incentivized BT participants following 30 weeks of intervention. We hypothesized that the parents and children randomized to BT+CM would have relatively improved weight outcomes. The secondary aims were to assess the following differences between the two groups: 1) study attendance and adherence, which were measured as (a) the percentage of dyads who completed the 30-week study follow-ups, (b) the percentage of dyads who attended each of the 30 study visits, 2) changes in fasting blood tests, and 3) changes in liver fibrosis and steatosis scores assessed non-invasively via liver transient elastography (TE). We predicted more significant improvements in the laboratory parameters and TE measurements in children randomized to BT+CM. We also present preliminary data on TE changes in obese youth participating in intensive weight management using dual CM.

## Materials and methods

Study Subjects

The research team aimed to enroll 20 obese youth, aged 12-18 years, and their parents. Obesity disproportionately affects families with social disparities [4]. Therefore, we focused on enrolling families receiving government-supported health insurance. Participants referred to an urban tertiary weight management program were evaluated between April 2017 and June 2018. Signed informed consent and assent were obtained from parents and youth, respectively. The study was conducted at the John R Oishei Children's Hospital and the Clinical and Translational Research Center, the University of Buffalo/ Jacobs School of Medicine and Biomedical Sciences in Buffalo, New York and was approved by the Institutional Review Board of the University at Buffalo. It was performed per the ethical standards of the 1975 Helsinki Declaration.

We excluded participants if the family lacked the capacity or was unwilling to download phone apps or send or receive phone messages via personal cellphone or data usage plans. Additional exclusion criteria included: 1) untreated or unstable depression identified by a behavioral specialist on the initial visit; and 2) the use of medications that affect appetite and mood, including metformin and antipsychotics.

Study Design and Procedures

When families attended their first clinic visit, they were screened based on the above criteria, and if appropriate, they received complete baseline medical and behavioral measures. A previously published, standardized "Traffic Light Diet" [12] and a 60-minute per day stretching and activity plan were explained, and the adolescent and the parent were given written instructions. The screening visit was followed by a four-week run-in phase during which the parent-child dyad came to the clinic weekly and completed daily food and activity records. Families who attended all four sessions and completed recording for at least 80% of the diet and activity records (six of seven days per week) were randomized (using a random number table) to a BT control group or a BT+CM group and followed for the next 24 weeks. We aimed to recruit 10 participants in each group for this pilot study.

Both groups attended weekly sessions where the parents and youth were weighed and seen by the attending physician (IM) and behavioral specialists (KC and ET). They provided counseling on health behavior changes. In addition, both groups were given a smart body scale with a Bluetooth-connected smartphone app. Youth and parents were asked to weigh themselves at home every other day and share the information with the research team. They received social reinforcement via text messaging for regular weight checks or if more than four days passed without a weigh-in. Such social reinforcers have been demonstrated to impact weight loss [13]. The BT+CM dyad received a monetary reward of $5 every week if they lost at least one pound; this amount escalated every two weeks up to a maximum amount of $10 per week if both participants in the dyad continued to 1) follow up in clinic weekly; 2) weigh themselves and share data with the research team per protocol; 3) completed a diet and activity record six of seven days per week; and 4) lost at least one pound weekly. Weight gain, either in the parent or youth or a failure to follow up in the clinic, resulted in a return to the initial contingency amount. The rationale for the escalation in reward was that sustained weight loss becomes progressively more challenging [14]. Further, successful CM interventions have demonstrated the efficacy of increasing reinforcement magnitude to maintain the desired behaviors [8, 9]. The BT group received a fixed amount for attending the treatment sessions. The BT control group acted as a robust control for BT+CM. It directly controls for the fact that providing baseline incentives can be energizing and can influence attendance independent of weight loss.

Data Collection

For all participants, weight and height were measured according to a standardized protocol. Weight was measured (with an electronic scale to the nearest 0.1 kg) at each clinic visit, and height (with a calibrated wall-mounted stadiometer to the nearest 0.1 cm) was measured at baseline for parents and youth with Tanner stage 5. The Tanner stage was assessed at the baseline by a single investigator (IM). Height was re-measured in growing children (Tanner stages 1-4) at weeks 18 and 30. BMI was calculated as kg/m2 for both parents and youth, and zBMI was obtained from the patient's standard electronic medical record (PowerChart®).

Laboratory Parameters

Blood tests were collected within two weeks of the initial visit and at week 30 and included fasting (10 to 12-hour) blood samples for lipid profiles (total cholesterol, low-density lipoprotein (LDL)-cholesterol, high-density lipoprotein (HDL)-cholesterol, triglyceride concentrations), glucose, insulin, aspartate aminotransferase (AST), alanine aminotransferase (ALT), and gamma-glutamyl transpeptidase (GGT) concentrations, and hemoglobin A1C percentage (HbA1c). All blood tests were performed at the institutional laboratory (Kaleida Health Department of Pathology and Laboratory Medicine, Buffalo, NY). The homeostasis model assessment of insulin resistance (HOMA-IR) was calculated as follows: fasting glucose (mmol/l) x fasting insulin (µIU/ml)/22.5. This tool correlates with insulin resistance as measured by the euglycemic-hyperinsulinemic clamp method [15]. In addition, we compared changes in laboratory parameters within and between groups following 30 weeks of intervention.

Liver TE (FibroScan®)

TE is a device that assesses liver stiffness and steatosis. It is based on shear wave velocity and an ultrasound coefficient of attenuation (Controlled Attenuation Parameter (CAP)) [16]. It has been used in adults to measure liver stiffness (LSM, expressed in kPa (kiloPascal)) and fat deposition (CAP, expressed in decibels per meter (dB/m)) non-invasively, with higher measurements correlating with increasing stiffness and steatosis. Liver stiffness is a surrogate for fibrosis, and elevated liver stiffness values correlate well with cirrhosis [17]. Trained technicians performed TE measurements at baseline and the end of the study period. A hepatologist and gastroenterologist supervised the assessments. CAP was calculated if the liver stiffness measurement was valid. CAP was measured with the M probe at 3.5 MHz at depths between 25 and 65 mm or with the XL at 3.5 MHz at depths between 35 and 75 mm per the manufacturer's instructions.

Food, Activity, and Home Weight Monitoring

MyFitnessPal® app was used to track food intake. Pacer pedometer was used for activity monitoring. Upon enrollment, the research team taught families how to use the phone apps. One electronic scale (Yolanda Precision Smart Body Scale with Bluetooth) was given per family after a successful run-in and randomization. All participants were required to weigh themselves every other day at home using the smart scale since frequent weight monitoring is associated with weight loss [18]. We used the compatible app to record parents' and children's weights and share these values with the research team. In addition, participants received standardized social reinforcement messages from the research team via a secure messaging app (WhatsApp®).

Statistical Analysis

Participants were randomized using a random number table. Data were expressed as mean ± standard deviation (SD) for continuous variables and frequency for categorical data. Separate linear mixed-effects regression models (adjusted for baseline z-BMI (child) or BMI (parent)) were used to assess differences in the trajectory of weight change from baseline to the 30-week follow-up (z-BMI for children and BMI for parents, respectively) between the BT and BT+CM groups. Linear mixed-effects models account for random missing data and the non-independence of observations. Cohen's d effect size was additionally calculated. When all appropriate data were available, we measured the changes in BMI, BIA, laboratory parameters, and TE changes in participants. Pearson's correlation was used to test the associations between the changes in BMI, BIA, laboratory parameters, and the measurement of TE changes. Analyses were performed using SPSS 21.0 (SPSS, Chicago, IL, USA) and Systat v.13 (Systat software, 2004). A p-value < 0.05 was considered statistically significant.

## Results

We screened 273 families, and 14 parent-child dyads agreed to participate. Work and school schedules and significant mental health disorders were barriers to participation. Five families dropped out during the four-week run-in phase, and the remaining nine dyads were randomized to either BT (n= 4) or BT+CM (n= 5) (Table 1). BT participants included one non-Hispanic white (NHW) male, one middle-east Asian male, and two males whose race or ethnicity was not reported. The BT+CM participants included three NHW females, one African American female, and one male; race and ethnicity were not reported. The parents included were 100% female (Table 1).

**Table 1:** Comparison of data of participants randomized to each group BT: behavioral therapy; CM: contingency management; BMI: body mass index; zBMI: BMI z-score; FM: total body fat mass determined by body impedance analysis; MM: total body muscle mass determined by body impedance analysis *Median and range

	Youth		Parent	
	BT (n= 4)	BT+CM (n= 5)	BT (n= 4)	BT+CM (n= 5)
Age (years)	14.5± 2.0	14.8± 1.5	41± 4.7	43.6± 6.9
Gender (M/F)	4:0	1:4	0:4	0:5
Tanner stage	4 (1-5)*	4 (3-5)*		
Weight (Kgs)	121.03± 38.9	139.4± 52.8	119.2± 20.2	100.4± 2.3
BMI	40.4±7.3	45.8±11.9	44.9±6.3	37±1.7
zBMI	2.7± 0.3	2.74± 0.4		
FM (Kilograms)	46.4±18.7	64.3± 36.1	56.8± 12.9	43.3± 2.9
MM (Kilograms)	70.5±21	71.1±18.8	59.4±10.1	54.3±1.2

Only one of the four BT dyads completed the 30-week study follow-up. The remainder of the three BT participants stopped participating at weeks 10, 11, and 28. Of the five BT+CM participants, four dyads completed the full 30-week follow-up, while the fifth participant ended participation at week 23.

All participants made progress in weight parameters over the 30-week follow-up. For the youth, the average weight decrease was 3.5±10.2 kilograms (kg), the zBMI decreased by 1±0.2, the total body fat mass decreased by 5.2±8.5 kg, and the total body muscle mass increased by 1±7.2 kg. The parents also lost weight; the average weight decreased by 4.9±8.4 kg, the BMI decreased by 1.9±3.3, the total body fat decreased by 6.7±6.8 kg, and the total body muscle mass decreased by 1.7±2.9 kg.

The main effect of the treatment group in the mixed effect models provides an estimate and standard error of differences in the magnitude of weight change. There was no main effect of the group for child z-BMI between the BT and BT+CM groups over the course of the study (β= 0.036, SE=0.033, p = 0.265). However, there was a greater rate of change in child zBMI in the BT group relative to the BT+CM group (group x time interaction: β = 0.005; SE = 0.001; p <0.01). For parent analyses, the group had a significant main effect on BMI, favoring the BT group (β= 1.902, SE=0.766, p = 0.013). There was also a greater rate of change in parent BMI for the BT group relative to the BT+CM group (group x time interaction: β = 0.085; SE = 0.010; P < 0.01). We observed a child and parent from one family in the BT group that lost substantially more weight than others. Hence, we ran separate analyses with this family's data removed. These analyses found no statistically significant main effect of group for child zBMI or parent BMI models. Further, the group-by-time interaction for child zBMI was no longer significant (p= 0.400), but the group-by-time interaction for parent BMI remained significant, favoring the BT group (p<0.01). BIA parameter changes did not show significant differences between groups in either youth or parents.

Compared to BT participants, the BT+CM participants had a trend towards higher rates of 30-week study completion and study-visit attendance at each of the 30 study visits. We noted large effect sizes for the 30-week study completion rate (Cohen's d=1.24) and study-visit attendance at each of the 30 study visits (Cohen's d =1.59) (Table 2).

**Table 2:** Comparison of study participation adherence BT: behavioral therapy; CM: contingency management * p > 0.05 **Cohen’s d effect size

	BT (n=4) *	BT+CM (n= 5) *	Effect size**
Percentage of participants completing 30- week follow up	25	80	1.24
Percentage of study-visit attendance at each of the 30 study visits	47.5	87.9	1.59

Blood test results were available at baseline for all youth. At the end of the study, results were available for all BT+CM youth and only one BT participant. The baseline fasting blood tests (HbA1c, glucose concentrations, HOMA-IR, total, LDL and HDL cholesterol, triglyceride, AST, ALT, and GGT concentrations) were comparable in both groups. At week 30, repeat tests did not change significantly in BT+CM participants. However, blood tests were available in only one BT participant at the end of the 30-week follow-up, which precluded comparison to baseline values and between groups (data not shown).

**Figure 1:**
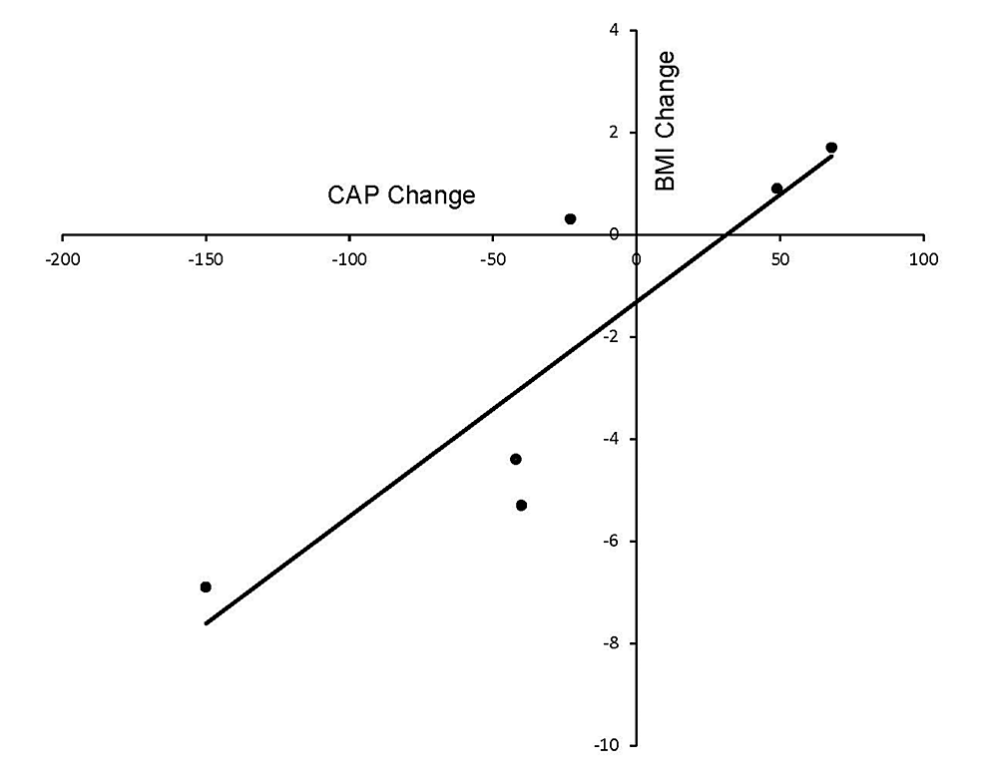
Association between the changes in body mass index (BMI, kg/m2) and controlled attenuation parameter (CAP, expressed in decibels per meter) measured by transient elastography in youth with obesity undergoing intensive weight management over 30 weeks Pearson’s correlation R= 0.98, R² = 0.79, p<0.001

**Figure 2:**
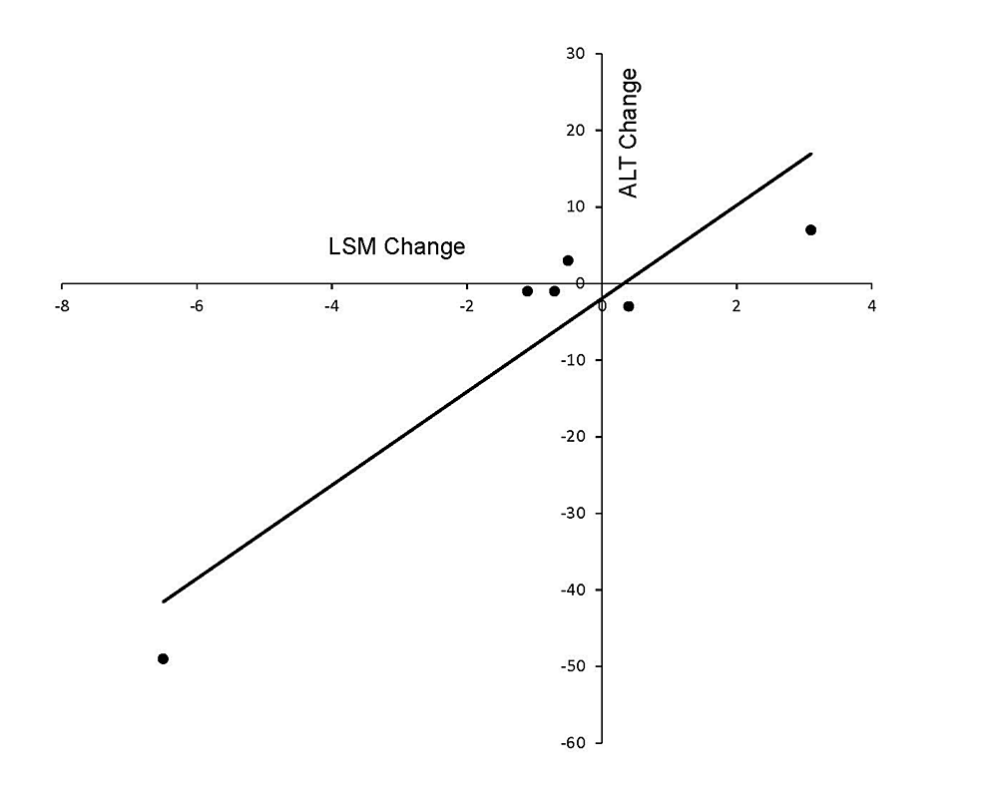
Association between the changes in alanine aminotransferase (ALT, units/L) and liver stiffness (LSM, expressed in KiloPascal) measured by transient elastography in youth with obesity undergoing intensive weight management over 30 weeks Pearson’s correlation R= 0.98, R² = 0.85, p=0.05

The changes in liver function tests and TE were assessed en bloc for all youth since we had follow-up data on only one out of four BT and all BT+CM participants. At baseline, AST (22.9±11.1 U/L), ALT (25.8±23.3 U/L), and GGT (22.9±10.3 U/L) were normal (n= 9). The TE parameters of youth were LSM= 6.2±3.4 kPa and CAP= 270.6±44.4 dB/m. At week 30, there was a trend towards lower AST (17.3±4.8 U/L), ALT (17.8±10.4 U/L), GGT (15.6±5.2 U/L), LSM= 4.8±1.4 kPa, and CAP= 250.3±54.4dB/m (n= 6). Changes in CAP correlated with changes in BMI (R2= 0.78, p< 0.001) (Figure 1), zBMI (R2= 0.72, p= 0.04), and LSM correlated with changes in ALT (R2= 0.85, p=0.005) (Figure 2).

 

.

## Discussion

The treatment of pediatric obesity is very challenging. Intensive family-based therapy remains the backbone of management. Due to the recalcitrant nature of the disease, maintaining healthy changes requires ongoing contact between families and their healthcare team to provide constant support and encouragement. However, improvements in weight and metabolic parameters may not be sufficient to motivate families facing socioeconomic challenges to continue their close follow-ups with their providers [19]. Therefore, more powerful behavioral economic strategies are required to maintain healthy outcomes.

CM is a tool showing promise in supporting adult and pediatric weight management strategies [10, 11]. However, weight management in youth is more effective when parents or guardians participate and the family tries to lose weight as a whole [20]. Therefore, we designed our novel dual CM model to encourage concurrent weight loss by rewarding the dyad only when the parent and the child lost weight together during their weekly clinic visits. The reward escalated every two weeks to a preset maximum limit if the parent and child continued to lose weight simultaneously. In addition to simultaneous weight loss, we hypothesized that the dual CM model would increase adherence to the weekly study visit attendance due to dual incentivization.

In the 30-week intensive weekly follow-up design, we anticipated difficulties in recruiting and retaining parent-child dyads. Not surprisingly, the one-year, time-limited study faced significant recruitment challenges. Of the initial 273 families approached, only 14 ultimately participated. Many parent-child dyads expressed an unwillingness to come for weekly visits, citing school or academic commitments for the child or work commitments for the parents. We also encountered significant mental health disorders in both the parent and child, precluding many parent-child dyads' participation. The mental health disorders included unstable depression, developmental delays, and antipsychotic medication use. Only one family reported the lack of a smartphone or tablet as a barrier to participation.

The intervention model showed positive benefits, and all participants showed a trend toward weight loss. Similar trends were also seen in the total body fat and muscle mass measurements. However, we could not demonstrate significant differences between groups, possibly due to our small sample size and the inability to maintain ongoing participation in the non-incentivized BT dyads.

Adding CM to BT appeared to improve participants' retention and adherence to the study protocols. In particular, the percentage of participants attending each of the 30 clinic visits and those completing the 30-week follow-ups appeared to be higher in BT+CM participants, with large effect sizes between groups. These results suggest that CM added to BT may be a powerful tool to modify behaviors. In addition, it likely encouraged both the parent and the child to participate in our intensive 30-week follow-up despite multiple socioeconomic challenges.

Limitations

We acknowledge the following limitations: First, we recruited participants from an urban tertiary weight management center, possibly representing those more engaged and seeking weight management assistance. Not surprisingly, all parent-child dyads had a weight-loss trend over the 30-week follow-up. Further, we noted that none of the BT+CM participants reached the preset maximum reward during the study visits. Either the parent or child would obviate the reward escalation. Some parent-child dyads appeared frustrated with the lack of a reward increase. Therefore, we may test an alternate CM model rewarding individual achievements with additional escalation for future dual or simultaneous goal achievements. Further, a community-based intervention may assess the effectiveness of this dual CM model in the general pediatric population.

A secondary objective of this study was to assess changes in hepatic stiffness and steatosis using novel technology in youth undergoing intensive weight management. NAFLD and non-alcoholic steatohepatitis (NASH) are obesity-associated comorbidities [21]. Liver biopsy is the "gold standard" for diagnosing NAFLD and NASH [22], but the need for an invasive procedure limits its utility for early NAFLD and NASH detection. On the other hand, NAFLD/NASH is often undetectable early by routine blood tests [23]. Therefore, early detection may promote early intervention to prevent disease progression. TE is increasingly used in adults to detect hepatic steatosis and fibrosis [24, 25]. Limited data in non-obese children show an age-dependent increase in LSM but not CAP [26]. Normative literature is sparse on children with obesity [26, 27]. This study provided preliminary data demonstrating the utility of TE in monitoring hepatic steatosis in youth with extreme obesity who were undergoing an intensive lifestyle intervention. Due to our small sample size, we assessed the data of all youth en bloc to analyze the association of the changes in liver function and the changes in TE parameters with weight loss. None of the youth recruited in the study had abnormal AST or ALT at baseline. Nonetheless, the liver enzyme concentrations showed improving trends after 30 weeks of intensive weight management, as well described in the literature [28].

TE measurements at baseline in our youth were comparable to normative parameters described in the literature [29]. Interestingly, the TE measurements in youth showed improving trends with intensive weight management. Unfortunately, our small sample size precluded the demonstration of statistically significant differences in the final TE measurements when compared to the baseline values. However, the univariate analysis indicated a strong association between the changes in CAP score and BMI changes. Additionally, ALT changes showed a strong association with changes in LSM scores. These results are similar to recent data published in Japan, where weight reduction was associated with improvements in CAP and LSM in children with NAFLD [30].

## Conclusions

Our pilot study could not demonstrate significant improvements in BMI changes when CM was added to family-based BT therapy in youth and their parents. The authors feel that the small sample size and challenges in maintaining long-term follow-up with the non-incentivized participants led to our inability to demonstrate the efficacy of BT+CM in improving weight outcomes over standard BT. However, we illustrated its utility in maintaining behaviors, including adherence to intensive follow-up protocols. Therefore, the effectiveness of BT+CM, when combined with the judicious use of technology, must be tested, for it may provide a new paradigm for arresting multigenerational obesity.

An interesting secondary outcome of the study is the preliminary data on TE changes in youth with obesity who are undergoing intensive weight management. Our data indicate that TE may be a valuable tool to non-invasively monitor fatty liver disease in clinical practice in youth with obesity, where routine blood tests provide limited information.
